# Correction: The association between RDW-to-platelet ratio and in-hospital mortality in critically ill stroke patients: A retrospective cohort study based on the eICU database

**DOI:** 10.1371/journal.pone.0352777

**Published:** 2026-06-30

**Authors:** Yu Chen, Xiangrong Yang, Minmin Lan, Xinghua Qin, Dongmei Yi, Lu Chen

There is an error in the affiliation of the authors. The correct affiliation is: Department of Neurology, Guangxi Hydroelectric Hospital, Nanning, Guangxi, China.
