# Supplementary material for: The association between RDW-to-platelet ratio and in-hospital mortality in critically ill stroke patients: A retrospective cohort study based on the eICU database
Source: PLoS One. 2026 Apr 17;21(4):e0344361. doi: 10.1371/journal.pone.0344361 (PMC13089741; doi:10.1371/journal.pone.0344361)
Supplement: S1 Table — (DOCX) [file pone.0344361.s001.docx]

**S1 table.Missing Data Analysis**

| **Variable** | **Complete (n)** | **Missing (n)** | **Missing (%)** | **Handling Method** |
| --- | --- | --- | --- | --- |
| ****Albumin, g/dL**** | 6,892 | 2,844 | 29.21 | missForest imputation |
| ****APACHE-IV score**** | 8,324 | 1,412 | 14.50 | missForest imputation |
| ****Total GCS score**** | 9,482 | 254 | 2.61 | missForest imputation |
| ****Blood urea nitrogen, mg/dL**** | 9,688 | 48 | 0.49 | missForest imputation |
| ****Ethnicity**** | 9,689 | 47 | 0.48 | missForest imputation |
| ****Creatinine, mg/dL**** | 9,691 | 45 | 0.46 | missForest imputation |
| ****Gender**** | 9,735 | 1 | 0.01 | missForest imputation |
| ****Age, years**** | 9,736 | 0 | 0.00 | Complete data |
| ****Mechanical ventilation**** | 9,736 | 0 | 0.00 | Complete data |
| ****Congestive heart failure**** | 9,736 | 0 | 0.00 | Complete data |
| ****Hypertension**** | 9,736 | 0 | 0.00 | Complete data |
| ****Diabetes mellitus**** | 9,736 | 0 | 0.00 | Complete data |
| ****Malignant tumor**** | 9,736 | 0 | 0.00 | Complete data |
| ****Sepsis**** | 9,736 | 0 | 0.00 | Complete data |
| ****Hemoglobin, g/dL**** | 9,736 | 0 | 0.00 | Complete data |
| ****Anticoagulant therapy**** | 9,736 | 0 | 0.00 | Complete data |
| ****Antiplatelet therapy**** | 9,736 | 0 | 0.00 | Complete data |

Variables with missing rate ≥40% were excluded from analysis. When any variable has missing rate ≥5%, all covariates are imputed using missForest algorithm regardless of their individual missing rates. Final sample size after processing: 9,736.

Abbreviations: GCS, Glasgow Coma Scale; APACHE, Acute Physiology and Chronic Health Evaluation.
