# Supplementary material for: The association between RDW-to-platelet ratio and in-hospital mortality in critically ill stroke patients: A retrospective cohort study based on the eICU database
Source: PLoS One. 2026 Apr 17;21(4):e0344361. doi: 10.1371/journal.pone.0344361 (PMC13089741; doi:10.1371/journal.pone.0344361)
Supplement: S2 Table — (DOCX) [file pone.0344361.s002.docx]

**S2 Table. Convergence diagnostics of the missForest imputation algorithm**

| **Iteration** | **Mean Delta* (%)** | **NRMSE Albumin (g/dL)** | **NRMSE APACHE-IV score** | **NRMSE Total GCS score** | **NRMSE Blood urea nitrogen (mg/dL)** | **PFC Ethnicity** |
| --- | --- | --- | --- | --- | --- | --- |
| 1 | 28 | 0.345 | 0.292 | 0.162 | 0.105 | 0.048 |
| 2 | 13 | 0.167 | 0.141 | 0.079 | 0.051 | 0.022 |
| 3 | 5.5 | 0.089 | 0.068 | 0.038 | 0.024 | 0.01 |
| 4 | 2.1 | 0.047 | 0.033 | 0.019 | 0.011 | 0.005 |
| 5 | 0.8 | 0.024 | 0.015 | 0.009 | 0.005 | 0.002 |
| 6 | 0.3 | 0.012 | 0.007 | 0.004 | 0.002 | 0.001 |

Notes: *Mean Delta represents the average relative change (%) in imputed values across all variables between two consecutive iterations. NRMSE = normalized root mean squared error (lower values indicate better imputation accuracy); PFC = proportion of falsely classified entries (for the categorical variable Ethnicity). The algorithm converged after a maximum of 6 iterations in all five imputation chains when the Delta fell below the pre-specified threshold of 0.01. Final OOB imputation errors were low, indicating high-quality imputation. These convergence results, together with the comparison of data distributions before and after imputation (see Table S2, all P > 0.05), confirm the reliability of the missForest approach.
