# Supplementary material for: The association between RDW-to-platelet ratio and in-hospital mortality in critically ill stroke patients: A retrospective cohort study based on the eICU database
Source: PLoS One. 2026 Apr 17;21(4):e0344361. doi: 10.1371/journal.pone.0344361 (PMC13089741; doi:10.1371/journal.pone.0344361)
Supplement: S3 Table — (DOCX) [file pone.0344361.s003.docx]

**S3 Table. Comparison of Original and Imputed Data Quality Assessment**

| **Variable** | **Original Data** | **Imputed Data** | **Test Statistic** | **P Value** |
| --- | --- | --- | --- | --- |
| ****Age, years**** | 67.06 ± 14.86 (n=9,736) | 67.06 ± 14.86 (n=9,736) | N/A | Complete data |
| ****Albumin, g/dL**** | 3.41 ± 0.67 (n=6,892) | 3.42 ± 0.58 (n=9,736) | F = 0.31 | 0.576 |
| ****APACHE-IV score**** | 54.74 ± 25.17 (n=8,324) | 54.73 ± 24.02 (n=9,736) | F = 0.02 | 0.894 |
| ****Total GCS score**** | 12.54 ± 3.55 (n=9,482) | 12.54 ± 3.51 (n=9,736) | F = 0.01 | 0.920 |
| ****Blood urea nitrogen, mg/dL**** | 22.05 ± 16.39 (n=9,688) | 22.03 ± 16.36 (n=9,736) | H = 0.08 | 0.777 |
| ****Creatinine, mg/dL**** | 1.27 ± 1.28 (n=9,691) | 1.27 ± 1.28 (n=9,736) | H = 0.03 | 0.862 |
| ****Gender**** |  |  | χ² = 0.00 | 0.984 |
| Male | 5,058 (52.0%) | 5,059 (52.0%) |  |  |
| Female | 4,677 (48.0%) | 4,677 (48.0%) |  |  |
| ****Ethnicity**** |  |  | χ² = 2.43 | 0.787 |
| African American | 1,188 (12.3%) | 1,189 (12.2%) |  |  |
| Asian | 208 (2.1%) | 209 (2.1%) |  |  |
| Caucasian | 7,326 (75.6%) | 7,371 (75.7%) |  |  |
| Hispanic | 397 (4.1%) | 397 (4.1%) |  |  |
| Native American | 45 (0.5%) | 45 (0.5%) |  |  |
| Other/Unknown | 525 (5.4%) | 526 (5.4%) |  |  |

Values are presented as mean ± SD for continuous variables and n (%) for categorical variables. Statistical comparisons were performed using one-way ANOVA (F-statistic) for normally distributed variables, Kruskal-Wallis test (H-statistic) for non-normally distributed variables, and Chi-square test (χ²) for categorical variables. All P values >0.05 indicate successful imputation without significant bias.

Abbreviations: SD, standard deviation; GCS, Glasgow Coma Scale; APACHE, Acute Physiology and Chronic Health Evaluation.
