# Supplementary material for: The association between RDW-to-platelet ratio and in-hospital mortality in critically ill stroke patients: A retrospective cohort study based on the eICU database
Source: PLoS One. 2026 Apr 17;21(4):e0344361. doi: 10.1371/journal.pone.0344361 (PMC13089741; doi:10.1371/journal.pone.0344361)
Supplement: S4 Table — (DOCX) [file pone.0344361.s004.docx]

**S4 Table . Results from Multiple Imputation Analysis**

| **Analysis** | **Imputation 1** | **Imputation 2** | **Imputation 3** | **Imputation 4** | **Imputation 5** | **Pooled Result** |
| --- | --- | --- | --- | --- | --- | --- |
| ****RDW-to-platelet ratio vs In-hospital mortality (Unadjusted)**** | 1.068 (1.056, 1.080) | 1.068 (1.056, 1.080) | 1.068 (1.056, 1.080) | 1.068 (1.056, 1.080) | 1.068 (1.056, 1.080) | 1.068 (1.056, 1.080) |
|  | P<0.001 | P<0.001 | P<0.001 | P<0.001 | P<0.001 | P<0.001 |
| ****RDW-to-platelet ratio vs In-hospital mortality (Adjusted)**** | 1.045 (1.031, 1.060) | 1.047 (1.033, 1.062) | 1.046 (1.032, 1.061) | 1.045 (1.031, 1.060) | 1.048 (1.034, 1.063) | 1.046 (1.032, 1.061) |
|  | P<0.001 | P<0.001 | P<0.001 | P<0.001 | P<0.001 | P<0.001 |

Results show odds ratios (95% CI) and P values for each individual imputation dataset (1-5) and the final pooled result using Rubin's rules. Adjusted model controlled for age, sex, comorbidities, laboratory parameters, and other clinically relevant variables.

Abbreviations: CI, confidence interval.
