# Supplementary material for: The association between RDW-to-platelet ratio and in-hospital mortality in critically ill stroke patients: A retrospective cohort study based on the eICU database
Source: PLoS One. 2026 Apr 17;21(4):e0344361. doi: 10.1371/journal.pone.0344361 (PMC13089741; doi:10.1371/journal.pone.0344361)
Supplement: Attachment — Submitted filename: Peer Review Report1.docx [file pone.0344361.s005.docx]

Peer Review Report

Manuscript Title: RDW-to-Platelet Ratio as a Predictor of In-Hospital Mortality in Critically Ill Stroke Patients: A Retrospective Cohort Study Based on the eICU Database

General Comments

This manuscript investigates the relationship between the Red Cell Distribution Width-to-Platelet Ratio (RPR) and in-hospital mortality in critically ill stroke patients using the eICU database. The study addresses a clinically relevant question regarding accessible biomarkers. However, the article has some methodological issues. Specifically, the rationale for covariate selection in the regression model is not sufficiently justified, and the distinction between "association" and "prediction" is blurred. Furthermore, crucial information regarding the calculation of the primary exposure (RPR) and the visual data (Figure 1) is missing or unclear. Given the retrospective nature of the study and the lack of external validation, the authors should refrain from overemphasizing "prediction" and focus on "independent association."

Recommendation: Major Revisions

Major Comments

1. Conceptual Framework: Association vs. Prediction

The title and conclusion frame RPR as a "Predictor" and discuss its "predictive value" (e.g., Abstract Line 14, Line 221). However, this is an explanatory retrospective study without an external validation cohort. Establishing a "predictor" requires demonstrating discrimination (e.g., C-statistic improvement) and calibration in a separate dataset.

Critique: The current analysis primarily establishes an independent association, not a validated clinical prediction tool.

Requirement: Please revise the Title, Abstract, and Discussion. Change terms like "Predictor" to "Association" (e.g., "Association between RPR and In-Hospital Mortality..."). The conclusion should state that elevated RPR is associated with mortality, rather than claiming it "forecasted" (Line 221) mortality or is ready for "risk stratification" (Line 28) without further validation.

2. Statistical Methodology: Rationale for Covariate Selection

Critique: It is unclear why specific variables were chosen for the initial pool.

Requirement:

Provide a clear justification for why specific variables (e.g., Albumin, BUN) were included as confounders.

3. Discrepancy in RPR Calculation and Units

There is a massive discrepancy between the methods and results regarding RPR values.

Issue: Methods (Line 78) define RPR = RDW(%) / Platelet(10^9^/L). A typical value would be

13/200=0.065. However, Table 1 shows values like "5.67" or "7.56" (approx. 100x larger).

Requirement: The authors must explicitly state the exact unit conversion used (e.g., did you multiply the ratio by 100?). Without this, the results are not reproducible, and the cut-off values are meaningless for other researchers.

4. Baseline Characteristics Table Presentation (Table 1)

Issue: The row "Sample size" in Table 1 contains the values 3,246 [1.16, 5.67], 3,245 [5.67, 7.56], etc. This formatting is confusing.

Clarification: It appears the numbers in brackets are the Range of RPR values for that tertile, not confidence intervals for the sample size.

Recommendation: Please create a separate row labeled "RPR Range" or "RPR Median [IQR]" to clearly present these values. Do not combine them with the sample size count (n).

5. Missing Figure 1 (Study Flowchart)

The manuscript references "Figure 1 Study Flow Diagram" (Line 68, Line 73, Line 145), but the figure itself is not visible in the provided document.

Requirement: A flow diagram is essential for assessing selection bias. It must clearly show the number of patients excluded at each step (especially how many were excluded due to missing RPR data vs. missing outcomes). Please ensure Figure 1 is included in the revised submission.

Minor Comments

Imputation of Albumin: Albumin has nearly 30% missing data (Line 115). While missForest is robust, 30% is a high proportion. Did the authors compare the characteristics of patients with vs. without albumin data to check for "Missing Not At Random" (MNAR) bias? A brief mention in the methods or supplement is needed.

Discussion of Mechanism: The discussion (Line 227) mentions "platelet count denotes coagulopathy". This is a simplification. In the context of stroke and inflammation, high platelets can be thrombotic (reactive thrombocytosis), while low platelets can be consumptive. RPR increases if Platelets decrease. The authors should discuss whether the high RPR in this cohort was driven primarily by high RDW, low Platelets, or both.

Stroke Subtype Heterogeneity: As mentioned in the Limitations, the study lumps Ischemic and Hemorrhagic stroke. While eICU lacks granular data, ICD-9 codes can often distinguish these. Please perform a sensitivity analysis stratifying by stroke type if possible, or discuss this limitation more prominently as a source of biological noise.

Mediation Analysis Interpretation: Mediation implies causality (RPR causes APACHE-IV change). Since they are measured simultaneously, this is conceptually weak. Please rephrase to suggest APACHE-IV "explains the variance" rather than definitively "mediates the effect."

Typos and Grammar:

Line 164: "linear trend . Mediation" (Remove extra space).

Line 26: "associated in-hospital mortality" -> "associated with in-hospital mortality".
