# Supplementary material for: The association between RDW-to-platelet ratio and in-hospital mortality in critically ill stroke patients: A retrospective cohort study based on the eICU database
Source: PLoS One. 2026 Apr 17;21(4):e0344361. doi: 10.1371/journal.pone.0344361 (PMC13089741; doi:10.1371/journal.pone.0344361)
Supplement: Attachment — Submitted filename: Response to Reviewers.docx [file pone.0344361.s007.docx]

Dear editors,

Thank you for your letter dated Jan 04 2026 concerning our manuscript titled "**The Association between RDW-to-Platelet Ratio and In-Hospital Mortality in Critically Ill Stroke Patients: A Retrospective Cohort Study Based on the eICU Database**" (Manuscript ID: PONE-D-25-61250). We appreciate the opportunity to revise and resubmit our work. We are grateful to you and the reviewers for the insightful comments and constructive criticism, which have significantly helped us improve the quality and clarity of our manuscript.

We have carefully considered all the points raised by the reviewers and have revised the manuscript accordingly. Our point-by-point responses to each comment are detailed below, with changes highlighted in the revised manuscript.

Yu Chen

**Reviewer #1:**

**1. The rationale for covariate selection in the regression model is not sufficiently justified**

Thank you for your thorough review of our manuscript and for the constructive criticism. We greatly appreciate your comment. We add the dedicated sentences of the covariate selection reasons. Showed as follow.

Covariate selection was guided by the following systematic principles: (1) Demographic variables (age, sex, race/ethnicity) were included as well-established independent prognostic factors for stroke patients; (2) Disease severity indicators (Acute Physiology and Chronic Health Evaluation IV [APACHE-IV] score) were selected for their recognized predictive value in critically ill patients for assessing acute physiological status; (3) Comorbidities (congestive heart failure, hypertension, diabetes mellitus, malignancy, sepsis) were incorporated based on extensive evidence linking these chronic conditions to adverse outcomes in stroke populations; (4) Therapeutic interventions (mechanical ventilation, anticoagulation, antiplatelet therapy) were included to capture clinical management intensity and underlying physiological derangement; (5) Laboratory parameters (albumin, blood urea nitrogen, hemoglobin, creatinine) were selected as they reflect nutritional status, renal function, anemia, and inflammation—each independently associated with mortality in critically ill patients across multiple studies in the MIMIC and eICU databases. All covariates were simultaneously included in the model to comprehensively adjust for potential confounding while minimizing the risk of over-adjustment, as verified by variance inflation factors (VIF<5 for all variables).

The selected covariates were chosen based on three principal considerations: First, substantial evidence from clinical literature—we systematically reviewed prognostic studies in critically ill stroke patients and confirmed that all included variables have been established as independent predictors of mortality in multiple investigations. Second, biological and clinical relevance—each variable either directly reflects disease biology (e.g., RDW as a marker of inflammation and bone marrow dysfunction) or represents an important clinical characteristic (e.g., APACHE-IV score quantifying illness severity). Third, data completeness and statistical feasibility with a sample size of 9,736 patients, the number of covariates was balanced to adequately control for confounding while maintaining statistical power. For variables with ≥5% missing values, we employed the missforest algorithm for multiple imputation, which outperforms traditional methods in handling complex missing data patterns and was validated through sensitivity analyses demonstrating robustness of results.

**2. The distinction between "association" and "prediction" is blurred. Given the retrospective nature of the study and the lack of external validation, the authors should refrain from overemphasizing "prediction" and focus on "independent association."**

We sincerely appreciate your valuable comments on our manuscript. Your feedback has helped us substantially improve the quality and precision of our work. We have carefully addressed all concerns, particularly regarding the important distinction between "association" and "prediction" in the context of our retrospective study design. Then we revised our manuscript.

**3. Furthermore, crucial information regarding the calculation of the primary exposure (RPR) and the visual data (Figure 1) is missing or unclear.**

Thank you for this important observation. We have substantially revised the manuscript to provide greater clarity regarding both the RPR calculation methodology. At the same time, we have re-designed our figure 1 and enhanced the Figure 1 legend. Showed as follow.

The primary exposure variable was the RDW-to-platelet ratio (RPR), defined as the initial Red Cell Distribution Width (RDW) value (expressed as a percentage, %) obtained within 24 hours of ICU admission divided by the platelet count (expressed as ×10⁹/L). For example, if a patient's RDW was 15.5% and platelet count was 250×10⁹/L, the RPR would be calculated as 15.5 ÷ 250 = 0.062.

Figure 1. Study Population Selection Flowchart.

The figure illustrates the sequential patient selection process from the initial eICU database (N=200,859) through application of inclusion and exclusion criteria to the final analytic cohort (N=9,736). The flowchart shows: (1) Initial screening of all eICU patients, with exclusion of non-stroke admissions (n=189,752); (2) Identification of stroke patients (n=11,107); (3) Exclusion of those with missing key variables including RDW-to-platelet ratio or in-hospital mortality data (n=1,371); and (4) Final classification of patients into outcome categories (survivors: n=8,375; in-hospital deaths: n=1,361). Abbreviation: eICU, eICU Collaborative Research Database; RDW, red cell distribution width.

**Reviewer 2#**

**1. This study adopts a retrospective cohort design, which could reveal the association between the red cell distribution width-to-platelet ratio (RPR) and in-hospital mortality in critically ill stroke patients, but cannot establish a causal relationship between them. There is a possibility of reverse causation: the progression of the patient's condition may lead to an increase in red cell distribution width (RDW) and a decrease in platelets, thereby elevating RPR, rather than elevated RPR directly increasing the risk of death. This potential logical relationship has not been effectively excluded.**

We agree that causal inference cannot be drawn from this observational study. We have revised the manuscript to consistently use language describing “association” rather than causation wherever appropriate.

**2. The study uses data from the 2014–2015 US multicenter eICU database, which is nearly a decade old. The research conclusions drawn based on outdated data have limited applicability in current clinical practice.**

We appreciate the thoughtful comments. In response to the issue raised about the use of historical eICU data (2014–2015) and its potential impact on the applicability of our findings to current practice, we have added limitation in our manuscript.

Notwithstanding compelling results, interpretive caution is warranted due to inherent limitations. Primarily, the retrospective observational design precludes causal inference regarding RPR and in-hospital mortality, with reverse causation (e.g., disease progression precipitating RDW escalation and platelet depletion) unexcluded[11]. Moreover, due to the 2014-2015 eICU data used, the data may not fully capture contemporary management of stroke in the ICU, potentially limiting generalizability to current clinical practice. Secondly...

**3. The database lacks key neurological information such as stroke subtype (ischemic or hemorrhagic), lesion location, and National Institutes of Health Stroke Scale (NIHSS) score. This makes it impossible to conduct stratified analysis for different subtypes of stroke patients and difficult to fully adjust for the impact of the degree of neurological impairment on prognosis, which may lead to confounding bias in the study results.**

Thank you for the valuable feedback. We acknowledge that the eICU-CRD data lack key neurological information (stroke subtype, lesion location, NIHSS), which limits our ability to perform stratified analyses by stroke subtype and to fully adjust for neurological impairment. Therefore, we have added our study shortcomings as limitations in the manuscript.

Notwithstanding compelling results, interpretive caution is warranted due to inherent limitations. Primarily, the retrospective observational design precludes causal inference regarding RPR and in-hospital mortality, with reverse causation (e.g., disease progression precipitating RDW escalation and platelet depletion) unexcluded[11]. Moreover, due to the 2014-2015 eICU data used, the data may not fully capture contemporary management of stroke in the ICU, potentially limiting generalizability to current clinical practice. Secondly, eICU's omission of stroke subtypes (ischemic vs. hemorrhagic), lesion loci, NIHSS scores, and imaging precludes granular neurological impairment adjustment. Third, reliance on admission-singleton RPR measurements neglects temporal fluctuations; Ye et al.[12] and Li et al.[15] affirmed that longitudinal platelet or RPR trajectories surpass baselines in sepsis prognostication, advocating serial assessments henceforth. Additionally, Tong et al.[17] highlighted RPR's independent predictivity in critically ill acute myocardial infarction yet cautioned comorbidity heterogeneity's bias amplification. Our U.S.-centric data may curtail generalizability amid racial and socioeconomic variances, particularly in Asian cohorts. Lastyly, the paucity of RPR-focused systematic reviews or meta-analyses necessitates prospective multicenter corroboration.

**4. The data are only derived from the US population, lacking samples from other ethnic groups (such as Asians, Africans, etc.) and regions. Thus, the applicability of the findings to non-US populations remains unclear.**

Thank you for your thoughtful feedback. In response to your guidance, we revised the manuscript to address the generalizability limitation due to the US-centric eICU-CRD data. Specifically, we added a limitations note clarifying that the dataset reflects care patterns in the United States during 2014–2015, which may limit applicability to non-US populations.

Notwithstanding compelling results, interpretive caution is warranted due to inherent limitations. Primarily, the retrospective observational design precludes causal inference regarding RPR and in-hospital mortality, with reverse causation (e.g., disease progression precipitating RDW escalation and platelet depletion) unexcluded[11]. Moreover, due to the 2014-2015 eICU data used, the data may not fully capture contemporary management of stroke in the ICU, potentially limiting generalizability to current clinical practice. Secondly, eICU's omission of stroke subtypes (ischemic vs. hemorrhagic), lesion loci, NIHSS scores, and imaging precludes granular neurological impairment adjustment. Third, reliance on admission-singleton RPR measurements neglects temporal fluctuations; Ye et al.[12] and Li et al.[15] affirmed that longitudinal platelet or RPR trajectories surpass baselines in sepsis prognostication, advocating serial assessments henceforth. Additionally, Tong et al.[17] highlighted RPR's independent predictivity in critically ill acute myocardial infarction yet cautioned comorbidity heterogeneity's bias amplification. Our U.S.-centric data may curtail generalizability amid racial and socioeconomic variances, particularly in non-US cohorts. Lastyly, the paucity of RPR-focused systematic reviews or meta-analyses necessitates prospective multicenter corroboration.

**5. The study only uses a single RPR measurement within 24 hours of admission as the exposure variable, without considering the dynamic changes of RPR during hospitalization. Relying solely on a single measurement may fail to fully reflect the patient's disease progression and real prognostic risk.**

Thank you for your review. The current analysis uses the initial RPR within 24 hours of ICU admission as the exposure variable to ensure comparability and avoid the complexity introduced by missing time-series data. According to your comments, We have added explicit descriptions of dynamic changes to the limitations and outlined specific plans for future research directions in the discussion.

Lastyly, the paucity of RPR-focused systematic reviews or meta-analyses necessitates prospective multicenter corroboration. In addition, although the results are robust, the study’s use of a single RPR measurement at admission remains a significant limitation. Future work should obtain multi-timepoint RPR data during hospitalization and apply time-varying analyses to evaluate the incremental prognostic value of RPR dynamics.

**6. The NIHSS score is an important indicator for evaluating the degree of neurological impairment and prognosis in stroke patients, but it was not included as a confounding factor in the adjustment, which may lead to overestimation or underestimation of the association between RPR and mortality.**

Thank you for your thoughtful feedback. In response to your guidance, we revised the manuscript to explicitly acknowledge the limitation that NIHSS data are not available in the eICU-CRD, which may lead to residual confounding in the association between RDW-to-platelet ratio (RPR) and in-hospital mortality. We added limitation note clarifying that the absence of NIHSS impedes stratified analyses by stroke subtype and full adjustment for neurological impairment.

Notwithstanding compelling results, interpretive caution is warranted due to inherent limitations. Primarily, the retrospective observational design precludes causal inference regarding RPR and in-hospital mortality, with reverse causation (e.g., disease progression precipitating RDW escalation and platelet depletion) unexcluded[11]. Moreover, due to the 2014-2015 eICU data used, the data may not fully capture contemporary management of stroke in the ICU, potentially limiting generalizability to current clinical practice. Secondly, eICU's omission of stroke subtypes (ischemic vs. hemorrhagic), lesion loci, NIHSS scores, and imaging precludes granular neurological impairment adjustment. Third, reliance on admission-singleton RPR measurements neglects temporal fluctuations; Ye et al.[12] and Li et al.[15] affirmed that longitudinal platelet or RPR trajectories surpass baselines in sepsis prognostication, advocating serial assessments henceforth. Additionally, Tong et al.[17] highlighted RPR's independent predictivity in critically ill acute myocardial infarction yet cautioned comorbidity heterogeneity's bias amplification. Our U.S.-centric data may curtail generalizability amid racial and socioeconomic variances, particularly in non-US cohorts. Lastyly, the paucity of RPR-focused systematic reviews or meta-analyses necessitates prospective multicenter corroboration.

**7. Ischemic stroke and hemorrhagic stroke differ significantly in pathophysiological mechanisms, treatment strategies, and prognosis, and the predictive value of RPR for mortality may vary between the two. However, the study did not perform subgroup analysis based on this key stratification factor, resulting in insufficient precision of the research** conclusions.

Thank you for your thoughtful feedback. In response to your guidance, we have explicitly added a limitations note under the manuscript’s Limitation section to reflect that NIHSS data are not available in the eICU-CRD, which may lead to residual confounding in the association between RDW-to-platelet ratio (RPR) and in-hospital mortality. We also note, per your suggestion, that the lack of stroke subtype information (ischemic vs hemorrhagic) precludes subgroup analyses and may affect precision.

Notwithstanding compelling results, interpretive caution is warranted due to inherent limitations. Primarily, the retrospective observational design precludes causal inference regarding RPR and in-hospital mortality, with reverse causation (e.g., disease progression precipitating RDW escalation and platelet depletion) unexcluded[11]. Moreover, due to the 2014-2015 eICU data used, the data may not fully capture contemporary management of stroke in the ICU, potentially limiting generalizability to current clinical practice. Secondly, eICU's omission of stroke subtypes (ischemic vs. hemorrhagic), lesion loci, NIHSS scores, and imaging precludes granular neurological impairment adjustment. Third, reliance on admission-singleton RPR measurements neglects temporal fluctuations; Ye et al.[12] and Li et al.[15] affirmed that longitudinal platelet or RPR trajectories surpass baselines in sepsis prognostication, advocating serial assessments henceforth. Additionally, Tong et al.[17] highlighted RPR's independent predictivity in critically ill acute myocardial infarction yet cautioned comorbidity heterogeneity's bias amplification. Our U.S.-centric data may curtail generalizability amid racial and socioeconomic variances, particularly in non-US cohorts. Lastyly, the paucity of RPR-focused systematic reviews or meta-analyses necessitates prospective multicenter corroboration.

**8.The study mentions using the missForest algorithm for multiple imputation to handle missing data, but it does not detail the specific parameter settings of the algorithm, the convergence test results during the imputation process, nor fully compare the differences in data distribution characteristics before and after imputation (only stating that there were no significant differences in key variables). This makes it difficult to evaluate the rationality and reliability of the imputation method.**

Thank you for your review. We have added the information in the methods part. Related tables are also showed in the supple tables.

To handle missing values in our dataset, we employed the missForest multiple imputation algorithm, which is based on random forests. This algorithm iteratively imputes missing data by building a random forest model for each variable with missing values, using all other variables as predictors to estimate the missing entries. We set the maximum number of iterations to 5 (maxiter = 5) to ensure convergence. For each random forest model constructed, we used 100 decision trees (ntree = 100). The number of variables randomly sampled as candidates at each split was set to the square root of the total number of predictor variables (mtry = sqrt(p), where p is the total number of predictors). To further enhance the stability of the imputation process, variables were imputed in decreasing order of the number of missing values (decreasing = TRUE). The convergence of the imputation process was confirmed by monitoring the changes in Out-of-Bag (OOB) error, with stabilization indicating model convergence. Detailed parameter settings and convergence diagnostics of the missForest algorithm are provided in Supplemental Table S4. The quality of the imputed data was assessed by comparing the distributions of key variables between the original and imputed datasets. As shown in Supplemental Table S2, no significant differences were observed in the distributions of key variables before and after imputation, confirming the rationality and reliability of the imputation method.

**9. The study uses a multivariable logistic regression model to analyze the association between RPR and in-hospital mortality, but only reports the odds ratio (OR) and confidence interval without assessing the model's calibration (e.g., Hosmer-Lemeshow test). Thus, the consistency between the model's predicted results and actual observed results cannot be determined.**

We appreciate the reviewers' valuable suggestions regarding the assessment of model calibration. We understand the importance of the Hosmer-Lemeshow test in evaluating the goodness of fit of predictive models. However, the core objective of this study is to explore the independent association between RPR and in-hospital mortality, aiming to quantify the effect size of RPR on the outcome (through adjusted OR) after adjusting for other confounding factors. Our analysis focuses on the accuracy of effect estimation, rather than developing a model for predicting individual risk. Therefore, we emphasize the use of a multivariate logistic regression model to effectively control for confounding factors and ensure the robustness of the OR. Given the association analysis nature of this study, we believe that the reported adjusted OR and its confidence intervals adequately reflect the strength of the association between RPR and in-hospital mortality. Nevertheless, we acknowledge the importance of calibration for future predictive model research and will consider it in future studies.

**10. The study only briefly mentions that RPR integrates pathophysiological processes related to inflammation and coagulation function, but does not deeply analyze the molecular mechanisms and pathophysiological pathways through which RPR affects patient prognosis in combination with the specific pathological mechanisms of stroke, resulting in insufficient theoretical depth of the research.**

Thank you for your comments. We have added this information in the discussion part.

... accounting for approximately 20.15% of the total effect, implying a pivotal intermediary role for disease severity in this biological pathway.

As an index combining red cell distribution width (RDW) and platelet count (PLT), RPR may participate in the pathophysiology of stroke through multiple pathways. Elevated RDW is often associated with systemic inflammatory responses, oxidative stress, and endothelial dysfunction, all of which exacerbate brain injury and neuronal apoptosis after stroke. Concurrently, platelet count directly reflects coagulation function; stroke patients often present with hypercoagulable states or platelet dysfunction, and changes in RPR may indicate an imbalance in the coagulation-fibrinolysis system, thereby affecting thrombus formation, reperfusion injury, or bleeding risk. Furthermore, RPR, as an indicator of red blood cell volume heterogeneity, may be related to red blood cell deformability and insufficient microcirculatory perfusion, particularly in ischemic brain tissue, which further aggravates tissue hypoxia and damage.

Our findings converge with multiple investigations leveraging MIMIC and eICU databases...
